# Mixed Viral Infections Circulating in Hospitalized Patients with Respiratory Tract Infections in Kuwait

**DOI:** 10.1155/2015/714062

**Published:** 2015-04-23

**Authors:** Sahar Essa, Abdullah Owayed, Haya Altawalah, Mousa Khadadah, Nasser Behbehani, Widad Al-Nakib

**Affiliations:** ^1^Department of Microbiology, Faculty of Medicine, Kuwait University, 24923 Safat, Kuwait; ^2^Department of Pediatrics, Faculty of Medicine, Kuwait University, 24923 Safat, Kuwait; ^3^Virology Unit, Mubarak Hospital, Ministry of Health, 24923 Safat, Kuwait; ^4^Department of Medicine, Faculty of Medicine, Kuwait University, 24923 Safat, Kuwait

## Abstract

The aim of this study was to determine the frequency of viral mixed detection in hospitalized patients with respiratory tract infections and to evaluate the correlation between viral mixed detection and clinical severity. Hospitalized patients with respiratory tract infections (RTI) were investigated for 15 respiratory viruses by using sensitive molecular techniques. In total, 850 hospitalized patients aged between 3 days and 80 years were screened from September 2010 to April 2014. Among the 351 (47.8%) patients diagnosed with viral infections, viral mixed detection was identified in 49 patients (14%), with human rhinovirus (HRV) being the most common virus associated with viral mixed detection (7.1%), followed by adenovirus (AdV) (4%) and human coronavirus-OC43 (HCoV-OC43) (3.7%). The highest combination of viral mixed detection was identified with HRV and AdV (2%), followed by HRV and HCoV-OC43 (1.4%). Pneumonia and bronchiolitis were the most frequent reason for hospitalization with viral mixed detection (9.1%). There were statistical significance differences between mixed and single detection in patients diagnosed with bronchiolitis (*P* = 0.002) and pneumonia (*P* = 0.019). Our findings might indicate a significant association between respiratory virus mixed detection and the possibility of developing more severe LRTI such as bronchiolitis and pneumonia when compared with single detection.

## 1. Introduction

The progress of molecular techniques for the identification of respiratory viruses allows for quick and specific diagnosis which is vital for the management of patients with respiratory tract infections (RTI) [1, 2]. Polymerase chain reaction (PCR) technology has been determined as an adequate tool for the identification of respiratory viruses as shown in many studies [3–5]. Respiratory virus infections represent a major public health problem because of their worldwide occurrence, ease of spread in the community, and considerable morbidity and mortality [6, 7]. The frequency of mixed respiratory viral detection varies from 10% to 30% in hospitalized children [8–11]. Several studies suggested an association between mixed detection and increase in the disease and/or clinical severity [9, 12–15]. Others propose the absence of a relationship between mixed respiratory detection and increase in the disease and/or clinical severity [16, 17].

Respiratory viruses such as human rhinovirus (HRV), respiratory syncytial virus (RSV), influenza A virus (FluA), influenza B virus (FluB), parainfluenza virus-1 (PIV-1), parainfluenza virus-2 (PIV-2), parainfluenza virus-3 (PIV-3), human coronavirus-OC43 (HCoV-OC43), human coronavirus-229E (HCoV-229E), and adenoviruses (AdV) have been recognized as causative agents of RTI [3, 18]. The panel of viruses determined responsible for RTI has been extended more by including more viruses such as human metapneumovirus (hMPV) [19, 20], HCoV-NL63 [21, 22], human bocavirus (Boca) [23, 24], human polyomavirus KI (KIV), and human polyomavirus WU (WUV) [25, 26].

The aim of this study was to determine the frequency of viral mixed detection in hospitalized patients with RTI and to evaluate the correlation between viral mixed detection and clinical severity.

## 2. Methods and Materials

### 2.1. Study Population

The study included 850 hospitalized children and adult patients with upper respiratory tract infections (URTI) or lower respiratory tract infections (LRTI) in Mubarak Al-Kabir Hospital, Kuwait. All patients hospitalized with RTI were screened during the period from September 2010 to April 2014. Specimens were collected in the hospital and stored at −70°C until processed in the Virology Unit, Faculty of Medicine, Kuwait University, to detect the presence of viral nucleic acids using PCR techniques. The age of the patients ranged between 3 days and 80 years. The majority of samples were collected during autumn and winter. Autumn in Kuwait is between September and November, and winter is between December and March.

### 2.2. Clinical Samples

Nasopharyngeal swab specimens for URTI and bronchoalveolar lavage for LRTI were collected after obtaining written informed consent from the hospitalized patients. Ethical permission to perform this research study was granted by the Health Science Center and Kuwait Institute for Medical Specialization (KIMS) Joint Committee of the Protection of Human Subjects in Research. Clinical data were collected from medical record using a uniform data collection form.

### 2.3. Molecular Detection of Respiratory Viruses

#### 2.3.1. Extraction Method

The nucleic acid extraction was done using the automated nucleic acid extraction method, MagNA Pure LC 2.0 (Roche Diagnostics Ltd., Rotkreuz, Switzerland). All 850 respiratory samples were extracted using the MagNA Pure LC Total Nucleic Acid Isolation Kit (Roche Applied Science, Mannheim, Germany) according to the manufacturer's instruction. The extraction resulting in 60 *μ*L eluates of viral nucleic acid was stored at −70°C until processing.

Determination of the presence of viral nucleic acids from respiratory viruses was performed as described before [4]. Briefly, a single PCR was used to detect adenovirus and parainfluenza virus-2 (PIV-2); duplex PCR was carried out to detect influenza A and B viruses; triplex PCR was carried out to detect respiratory syncytial virus (RSV) and parainfluenza viruses (PIV) 1 and 3, and another triplex PCR was performed to detect human rhinovirus and human coronavirus-229E and coronavirus-OC43.

#### 2.3.2. PCR and RT-PCR for the Detection of Newly Discovered Respiratory Viruses

Upon extraction of nucleic acids from clinical specimens, determination of the presence of hMPV RNA was performed using primers described by Ordás et al. [27]. HCoV-NL63 RNA was detected using primers described by Moës et al. [28], and Boca DNA was detected using primers described by Allander et al. [23]. The primers used to detect KIV and WUV DNA were previously described by Allander et al. and Bialasiewicz et al. [25, 26].

#### 2.3.3. PCR and RT-PCR Conditions

The RT step was performed for 60 min at 37°C in a 10 *μ*L reaction volume containing 1X GeneAmp RNA PCR buffer, 5 *μ*L of 25 mM MgCl_2_, 1 mM of each deoxynucleoside triphosphate, 2.5 *μ*M of random hexamers, 0.5 *μ*L RNase inhibitor, 3 *μ*L of viral nucleic acid, and 2.5 U/*μ*L reverse transcriptase enzyme (GeneAmp RNA Core Kit; Applied Biosystems, Chicago, IL). Following heat inactivation of the reverse transcriptase at 90°C for 5 min, the entire reaction mixture was used for PCR in a total volume of 50 *μ*L. The reaction mixture composition was 2 mM MgCl_2_ solution, 1X PCR buffer containing 0.02 pg of each forward and reverse primer, and 0.05 *μ*L of 5 U/*μ*L Ampli Taq DNA Polymerase (Thermo Fisher Scientific, Pittsburgh, USA). PCR was performed as follows: 94°C denaturation for 1 min, followed by 40 cycles of denaturation at 94°C for 30 sec, annealing at 50°C for 30 sec and elongation at 72°C for 30 sec, and a final extension at 72°C for 7 min. Water was used instead of nucleic acids as a negative control. The specificity of the PCR was established for each PCR format using a panel of ATCC reference viruses to check for cross-reactivity to old respiratory viruses. DNA templates (110–140 bp, Thermo Scientific) encompassing the annealing sites of the primers and probes were used as positive controls for the detection of nucleic acid from HCoV-NL63, hMPV, Boca, WUV, and KIV.

### 2.4. Statistical Analysis

Data analysis was performed using the Statistical Package for the Social Sciences (SPSS version 20.0, IBM Corp, Armonk, NY, USA). The descriptive statistics of the continuous variables were compared using a nonparametric Mann-Whitney *U* test or Kruskal-Wallis test. For the categorical variables, a Chi-square or Fisher's exact test or *Z*-test was applied to test the difference between proportions or to assess whether any association existed between the proportions. The two-tailed probability value *P* < 0.05 was considered statistically significant.

## 3. Results

From the overall number of 850 hospitalized patients three hundred fifty one patients (47.8%) were diagnosed with viral respiratory infections, 210 (59.8%) of them were males and 141 (40.2%) were females. Results show that from the 351 patients 408 viruses were detected.  Table 1 shows that HRV was the most detected virus in clinical respiratory specimens of patients with respiratory symptoms (41.6%), followed by FluA (15.1%), RSV (13.1%), and HCoV-OC43 (12.3%). Among the 351 hospitalized patients viral mixed detection was detected in 49 patients (14%). HRV was the most common virus associated with mixed detection (7.1%), followed by AdV (4%), HCoV-OC43 (3.7%), RSV (3.1%), and FluA (2.8%) (Table 1).

It was interesting to note that four patients had triple viral mixed detection. The first patient was infected with Boca, HCoV-OC43, and HRV, the second patient was diagnosed with WUV, Boca, and HCoV-229E, the third one was infected with HCoV-OC43, FluA, and HRV, and the fourth patient was infected with KIV, RSV, and hMPV.

Table 2 shows the frequency of viral mixed detection among the 49 patients. The highest combination of viral mixed detection was identified with HRV and AdV in 7 patients (2%), followed by HRV and HCoV-OC43 in 5 patients (1.4%), and HRV and FluA in 4 patients (1.1%).

From the 49 (14%) patients with mixed detection, 33 (9.4%) of them were males (32 patients (9.1%) with double detection and one patient (0.3%) with triple detection), and 16 (4.6%) were females (14 patients (4%) with double detection and 2 patients (0.6%) with triple detection) (Table 3).

In total, 20 of the 49 (5.7%) patients with viral mixed detection were aged <1 years (18 patients (5.1%) with double detection and 2 patients (0.6%) with triple detection), 17 patients (4.8%) were 1–14 years (16 patients (4.6%) with double detection and one patient (0.3%) with triple detection), and 12 patients (3.4%) were ≥15 with double viral detection (Table 3). Overall, the majority of viral mixed detection, reaching 8.5% (*n* = 30), was among children ≤5 years of age.  Table 4 shows the distribution of median age, range, and IQ of patients with mixed detection for each virus. The median age was <1 years for Boca, HCoV-OC43, and RSV whereas for WU, AdV, FluA, PIV-3, HRV, and hMPV it was 1–11.5 years. Furthermore, for the rest of the respiratory viruses KI, HCoV-229E, and PIV-1 it was ≥15 years of age.

Mixed viral detection was identified in 17 patients (4.8%) with pneumonia (15 patients (4.3%) with double viral detection and 2 patients (0.6%) with triple detection), 15 patients (4.8%) with bronchiolitis (14 patients (4%) with double viral detection and one patient (0.3%) with triple detection), 10 patients (2.8%) with URTI all suffered from double viral detection, and 7 patients (2%) with respiratory distress (RD) all suffered from double viral mixed detection (Table 3). the majority of infections by the investigated respiratory viruses affected the lower respiratory tract (39 patients or 11.1%) rather than the upper respiratory tract (10 patients, or 2.8%). Pneumonia and bronchiolitis were the most frequent reason for hospitalization with viral mixed detection (32 patients or 9.1%).  Table 1 compares the clinical manifestation of patients with mixed and single viral detection. There were statistical significance differences between mixed and single detection in patients diagnosed with bronchiolitis (*P* = 0.002) and pneumonia (*P* = 0.019).

The majority (32 patients or 9.1%) of hospitalized patients were admitted to wards, followed by pediatric intensive care unit (PICU) (11 patients or 3.1%), and intensive care unit (ICU) (6 patients or 1.7%) (Table 3).

**Figure 1:**
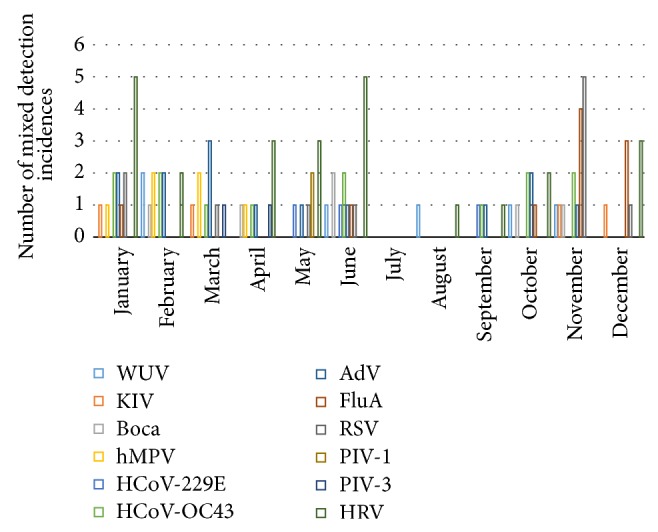
Monthly distribution of viral mixed detection.

The peak incidence of viral mixed detection was identified during the month of November (15 incidences of detection or 14.7%) followed by January and June (14 incidences of detection each or 13.7%). The lowest incidence was detected during the month of August (2 incidences of detection or 2%) and no viral mixed detection was identified during the month of July (Figure 1).

## 4. Discussion

The usage of molecular techniques for viral infections has improved the identification of mixed viral detection in a single sample [29]. In this study, we assessed the incidence of viral mixed detection in Kuwait during three and a half consecutive years, September 2010 to April 2014 by PCR techniques in hospitalized children and adults with URTI and LRTI. The overall prevalence of viral mixed detection in Kuwait among hospitalized patients with RTI was 14%. The frequency of mixed viral detection was approximately 8% higher in LRTI than in URTI. From the published studies that use molecular diagnostics to report respiratory viral mixed detection, no other studies match our study population (children and adults) or clinical presentation (URTI and LRTI). A community-based study in Jinan, China, of 720 samples from inpatient and outpatients with RTI during a one-year period identified viral mixed detection in 95 samples (13.19%). Also, in this study the virus positive rate was approximately 20% higher in LRTIs than in URTIs [9]. In a recent study 48% (140/292) of the samples from hospitalized children and adults with acute LRTI, viral mixed detection, were observed in 8% (22/292) of the samples [10]. In another recent study of 131 samples from children aged 0–8 with acute RTI, 19 (14.5%) were identified with mixed viral detection [11].

The three principal pathogens involved in mixed viral detection were HRV, AdV, and HCoV-OC43. Similar results were reported, where they identified HRV, AdV, and HCoV-OC43 as the leading viruses involved in mixed detection [30]. Other studies reported different groupings of leading viruses involved in mixed detection. Recent studies reported RSV, HRV, and AdV as the leading viruses involved in mixed detection among children [8, 31] and among children/adults [10]. In another study the most prevalent viruses involved in mixed detection among children with RTI were HRV, PIV, and Flu viruses [9]. These differences may be attributed to the panel of respiratory viruses tested, regional or environmental variability and the difference of the virus detection techniques.

Out of the 49 virally coinfected patients, 45 (12.8%) suffer from double viral detection and 4 (1.1%) triple viral detection. In an epidemiological study from Korea the mixed viral detection analysis showed 17.1% of double detection and 1.8% of triple detection, which is higher than our result probably due to the fact that they tested larger sample size and we tested a larger panel of viruses [30]. Another study also reported double 20.3% and triple 3.9% viral detection among children with RSV infection [8]. The most frequently detected combinations were HRV/AdV, HRV/HCoV-OC43, and HRV/FluA. The combination of HRV/AdV is the leading combination; this finding is directly comparable with those from previous reports [8, 30]. In this study, the majority of viral mixed detection was among children <1 years of age (20 patients or 5.7%). This is comparable with other recent studies [8–10, 31]. This may be due to an immature immune system of the infants and the absence of earlier exposure to respiratory viruses which could increase their susceptibility to a mixed infection [12].

In this study virus mixed detection was not identified between RSV and hMPV although a number of studies have found hMPV and RSV coinfection rates of approximately ~5–14% [20, 32, 33]. However, in a study conducted in Netherlands in hospitalized children with LRTI, no virus coinfection between RSV and hMPV was detected [34].

As shown in Table 2, HCoV-OC43 positive patients were most commonly coinfected with HRV and RSV. In a study conducted in China from 2006 to 2009 aimed to assess the overall prevalence of 10 respiratory viruses in children with acute LRTI, coronaviruses-positive samples were most commonly coinfected with HRV and RSV [35]. Similar data describing a high rate of mixed detection of coronaviruses with RSV has also been previously described [36, 37].

Since the first identification of KIV and WUV, their viral sequences have been identified globally in respiratory samples from patients with RTI [38–41]. However WUV and KIV were found at similar rates in control individuals without respiratory diseases so the association between these polyomaviruses and respiratory diseases remains hypothetical [38, 40, 42]. A mixed detection rate of 74% has been identified for KIV and rates stretching from 68 to 79% for WUV [39–41]. In this study, hospitalized patients with a single WUV detection were diagnosed with bronchitis, bronchiolitis, and pneumonia (Table 1). In a study in Southern China, hospitalized children with a single WUV detection presented with cough, moderate fever, and wheezing and they were also diagnosed with pneumonia, bronchiolitis, URTI, and bronchitis. These findings suggest that polyomavirus can cause URTI and LRTI [43]. In another study assessing the incidence and viral load of WUV and KIV in respiratory samples from immunocompromised and immunocompetent children revealed that the prevalence of WUV and KIV is similar in immunocompromised patients compared with that of the immunocompetent population [44]. Nevertheless these data have to be confirmed in further studies.

Several studies have shown that Boca detection tends to be associated with other respiratory viruses such as HRV, AdV, and RSV [23, 35, 45]. In this study Boca virus mixed detection was identified with HRV, HCoV-OC43, HCoV-229E, and AdV (Table 2). Persistent viral shedding and high frequency of mixed detection have led to an argument over its role as a true pathogen [42, 46]. Other studies confirmed that Boca virus is most probably the cause of RTI if the patient has a single detection and high viral load in clinical samples [45, 47]. In this study, our patients who were diagnosed with a single Boca virus detection suffered from both URTI and LRTI (Table 1). Nevertheless, despite this debate it is becoming increasingly obvious that Boca virus is an important respiratory virus [48].

Our findings might indicate an association between respiratory virus mixed detection and the possibility of developing more severe LRTI such as bronchiolitis (*P* = 0.002) and pneumonia (*P* = 0.019) when compared with single detection. The relationship between mixed viral detection and disease/clinical severity is debatable. Earlier studies have reported that mixed detection with respiratory viruses increased the risk of hospitalization and pneumonia [8, 9, 13, 14], while other studies reported no association between mixed detection and disease/clinical severity [16, 49]. However, despite the availability of sensitive molecular assays, reports are still controversial concerning the role of mixed detection in the disease/clinical severity in comparison to single detection. A number of theories have been proposed to explain the association between mixed respiratory virus detection and RTI severity; these theories include alteration of immune responses after the primary infection [50, 51] and host vulnerability to multiple viruses [15].

The seasonal incidence of mixed viral detection was detectable throughout the year except for the month of July, with the peak incidence during the months of January, June, and November (43 incidences of detection or 42.1%).

In summary, our findings may indicate that viral mixed detection in patients with RTI is not uncommon and that mixed detection may increase the clinical severity of patients with pneumonia or bronchiolitis. Further investigations are necessary to investigate the determinants of disease severity in viral mixed detection in RTI.

Although this study has several limitations like the lack of study controls (matched hospitalizations without RTI necessary to estimate attributable disease), difference in RT-PCR sensitivity/specificity among targeted pathogens, and lack of systematic testing for potential bacterial pathogens, viral loads were not detected but these data provide representative results of mixed respiratory viral detection in Kuwait.

## Figures and Tables

**Table 1:** Clinical manifestations of patients with mixed and single viral detection for twelve respiratory viruses.

Viruses	URTI		Bronchitis		Bronchiolitis		Pneumonia		LRTI		Total		*L* ^*L*^*L*^^Total (%)^∗∗^
	Mixed detection	Single detection	Mixed detection	Single detection	Mixed detection	Single detection	Mixed detection	Single detection	Mixed detection	Single detection	Mixed detection	Single detection	Viral detection
WUV	2	0	0	3	2	3	2	1	4	7	6 (1.7)	7 (2)	13 (3.7)
KIV	1	1	1	1	1	0	1	0	3	1	4 (1.1)	2 (0.6)	6 (1.7)
Boca	0	5	2	3	3	5	1	0	6	8	6 (1.5)	13 (3.7)	19 (5.4)
HCoV-OC43	1	9	1	13	4	3	7	5	12	21	13 (3.7)	30 (8.5)	43 (12.3)
HCoV-229E	0	1	0	5	2	5	1	0	3	10	3 (0.9)	11 (3.1)	14 (4)
AdV	2	4	3	6	4	3	5	3	12	12	14 (4)	16 (4.6)	30 (8.5)
FluA	4	5	2	11	3	8	1	19	6	38	10 (2.8)	43 (12.3)	53 (15.1)
RSV	3	9	2	0	3	19	3	7	8	26	11 (3.1)	35 (10)	46 (13.1)
PIV-3	1	2	0	2	1	2	0	2	1	6	2 (0.6)	8 (2.3)	10 (2.8)
HRV	2	5	2	39	8	45	13	32	23	116	25 (7.1)	121 (33.9)	146 (41.6)
PIV-1	0	1	0	1	1	1	1	1	2	3	2 (0.6)	4 (1.1)	6 (1.7)
hMPV	4	5	1	8	0	3	1	0	2	11	6 (1.7)	16 (4.6)	22 (6.3)

Total (%)^†^	20 (19.6)	47 (15.4)	14 (13.7)	92 (30.1)	32 (31.4)^∗^	97 (31.7)	36 (35.3)^∗^	70 (22.9)	82 (80.3)	259 (84.6)	102 (25)	306 (75)	408

^†^Number in parentheses represents the number of detection incidences (single or mixed) in relation to number of total (single or mixed) detection incidences in percent.

^∗∗^Number in parentheses represents the number of detection incidences in relation to the total number of patients (*n* = 351) in percent.

^∗^Statistical significance differences between single and mixed detection in patients diagnosed with bronchiolitis and pneumonia *P* < 0.05.

**Table 2:** Frequency of viral mixed detection for twelve respiratory viruses.

Viruses	WUV	KIV	Boca	HCoV-OC43	HCoV-E229	AdV	FluA	RSV	PIV-3	HRV	P1V-1	hMPV
WUV												
KIV	—											
Boca	1	—										
HCoV-OC43	—	1	1									
HCoV-229E	1	—	1	—								
AdV	—	1	1	3	—							
FluA	1	1	—	—	—	1						
RSV	1	1	—	3	—	—	2					
PIV-3	—	—	—	—	—	—	—	1				
HRV	1	—	2	5	1	7	4	3	—			
P1V-1	—	—	—	—	—	—	—	—	—	1		
hMPV	1	—	—	—	—	1	1	—	1	1	1	

Total^∗^	6	4	6	13	3	14	10	11	2	25	2	6

Data are number of samples positive for viral mixed detection.

^∗^Total number of mixed detection for each virus.

**Table 3:** Distribution of patients with undetected, single, and mixed detection in relation to age, gender, and hospital admission.

	Undetected	Single detection	Mixed detection
	Patient number 499 (%)^∗^	Patient number 302 (%)^∗∗^	Patient number 49 (%)^∗∗^
Age (yrs)			
<1	90 (18)	148 (42.2)	20 (5.7)
1–14	144 (29)	66 (18.8)	17 (4.8)
≥15	265 (53.1)	88 (25.1)	12 (3.4)
Gender			
Male	276 (55.3)	177 (50.4)	33 (9.4)
Female	223 (44.7)	125 (35.6)	16 (4.6)
Hospital Unit			
ICU	184 (36.9)	36 (10.3)	6 (1.7)
PICU	175 (35.1)	91 (30)	11 (3.1)
Ward	139 (27.9)	175 (50)	32 (9.1)

^∗^Number in parentheses represents the number of undetected viral infections in relation to the total number of patients (*n* = 499) in percent.

^∗∗^Number in parentheses represents the number of detected viral infections in relation to the total number of patients (*n* = 351) in percent.

ICU: Intensive care unit.

PICU: Pediatric intensive care unit.

**Table 4:** Age distribution of respiratory virus found in mixed detection.

Virus	Median age^∗^	Range	IQ
WUV	11.5	3–68	4–35
KIV	19.5	1–45	1–43
Boca	0	0–3	0–2
HCoV-OC43	0.5	0–38	0–7
HCoV-229E	50	3–80	3–65
AdV	4	0–70	0–16
FluA	4.5	0–60	0–45
RSV	0	0–24	0-1
PIV-3	2	0–80	0–24
HRV	1	0–80	0–19
PIV-1	36	33–39	33–36
hMPV	1	0–68	0–21

^∗^Median age = 0 (zero was coded for <1 year).

IQ: Interquartile range.

## References

[1] Ivaska L., Niemelä J., Heikkinen T., Vuorinen T., Peltola V. (2013). Identification of respiratory viruses with a novel point-of-care multianalyte antigen detection test in children with acute respiratory tract infection. *Journal of Clinical Virology*.

[2] Lévêque N., Renois F., Andréoletti L. (2013). The microarray technology: facts and controversies. *Clinical Microbiology and Infection*.

[3] Bierbaum S., Forster J., Berner R. (2014). Detection of respiratory viruses using a multiplex real-time PCR assay in Germany, 2009/10. *Archives of Virology*.

[4] Khadadah M., Essa S., Higazi Z., Behbehani N., Al-Nakib W. (2010). Respiratory syncytial virus and human rhinoviruses are the major causes of severe lower respiratory tract infections in Kuwait. *Journal of Medical Virology*.

[5] Li J., Qi S., Zhang C. (2013). A two-tube multiplex reverse transcription PCR assay for simultaneous detection of sixteen human respiratory virus types/subtypes. *BioMed Research International*.

[6] Denny F. W. (1995). The clinical impact of human respiratory virus infections. *The American Journal of Respiratory and Critical Care Medicine*.

[7] Karaivanova G. M. (1995). Viral respiratory infections and their role as public health problem in tropical countries (review). *African Journal of Medicine and Medical Sciences*.

[8] Harada Y., Kinoshita F., Yoshida L. M. (2013). Does respiratory virus coinfection increases the clinical severity of acute respiratory infection among children infected with respiratory syncytial virus?. *The Pediatric Infectious Disease Journal*.

[9] Lu Y., Wang S., Zhang L. (2013). Epidemiology of human respiratory viruses in children with acute respiratory tract infections in Jinan, China. *Clinical and Developmental Immunology*.

[10] Sentilhes A.-C., Choumlivong K., Celhay O. (2013). Respiratory virus infections in hospitalized children and adults in Lao PDR. *Influenza and other Respiratory Viruses*.

[11] Tecu C., Mihai M. E., Alexandrescu V. I. (2013). Single and multipathogen viral infections in hospitalized children with acute respiratory infections. *Roumanian Archives of Microbiology and Immunology*.

[12] Drews A. L., Atmar R. L., Glezen W. P., Baxter B. D., Piedra P. A., Greenberg S. B. (1997). Dual respiratory virus infections. *Clinical Infectious Diseases*.

[13] Franz A., Adams O., Willems R. (2010). Correlation of viral load of respiratory pathogens and co-infections with disease severity in children hospitalized for lower respiratory tract infection. *Journal of Clinical Virology*.

[14] Foulongne V., Guyon G., Rodière M., Segondy M. (2006). Human metapneumovirus infection in young children hospitalized with respiratory tract disease. *Pediatric Infectious Disease Journal*.

[15] Semple M. G., Cowell A., Dove W. (2005). Dual infection of infants by human metapneumovirus and human respiratory syncytial virus is strongly associated with severe bronchiolitis. *Journal of Infectious Diseases*.

[16] Martin E. T., Kuypers J., Wald A., Englund J. A. (2012). Multiple versus single virus respiratory infections: viral load and clinical disease severity in hospitalized children. *Influenza and Other Respiratory Viruses*.

[17] Wang Y., Ji W., Chen Z., Yan Y. D., Shao X., Xu J. (2014). Comparison of severe pneumonia caused by human metapneumovirus and respiratory syncytial virus in hospitalized children. *Indian Journal of Pathology and Microbiology*.

[18] Karadag-Oncel E., Ciblak M. A., Ozsurekci Y., Badur S., Ceyhan M. (2014). Viral etiology of influenza-like illnesses during the influenza season between December 2011 and April 2012. *Journal of Medical Virology*.

[19] van den Hoogen B. G., de Jong J. C., Groen J. (2001). A newly discovered human pneumovirus isolated from young children with respiratory tract disease. *Nature Medicine*.

[20] Williams J. V., Harris P. A., Tollefson S. J. (2004). Human metapneumovirus and lower respiratory tract disease. *The New England Journal of Medicine*.

[21] Pyrc K., Berkhout B., van der Hoek L. (2007). The novel human coronaviruses NL63 and HKU1. *Journal of Virology*.

[22] van der Hoek L., Pyrc K., Jebbink M. F. (2004). Identification of a new human coronavirus. *Nature Medicine*.

[23] Allander T., Jartti T., Gupta S. (2007). Human bocavirus and acute wheezing in children. *Clinical Infectious Diseases*.

[24] Allander T., Tammi M. T., Eriksson M., Bjerkner A., Tiveljung-Lindell A., Andersson B. (2005). Cloning of a human parvovirus by molecular screening of respiratory tract samples. *Proceedings of the National Academy of Sciences of the United States of America*.

[25] Allander T., Andreasson K., Gupta S. (2007). Identification of a third human polyomavirus. *Journal of Virology*.

[26] Bialasiewicz S., Whiley D. M., Lambert S. B., Gould A., Nissen M. D., Sloots T. P. (2007). Development and evaluation of real-time PCR assays for the detection of the newly identified KI and WU polyomaviruses. *Journal of Clinical Virology*.

[27] Ordás J., Boga J. A., Alvarez-Argüelles M. (2006). Role of metapneumovirus in viral respiratory infections in young children. *Journal of Clinical Microbiology*.

[28] Moës E., Vijgen L., Keyaerts E. (2005). A novel pancoronavirus RT-PCR assay: frequent detection of human coronavirus NL63 in children hospitalized with respiratory tract infections in Belgium. *BMC infectious diseases*.

[29] Kuypers J., Wright N., Ferrenberg J. (2006). Comparison of real-time PCR assays with fluorescent-antibody assays for diagnosis of respiratory virus infections in children. *Journal of Clinical Microbiology*.

[30] Kim J. K., Jeon J.-S., Kim J. W., Rheem I. (2013). Epidemiology of respiratory viral infection using multiplex RT-PCR in Cheonan, Korea (2006–2010). *Journal of Microbiology and Biotechnology*.

[31] Cantais A., Mory O., Pillet S. (2014). Epidemiology and microbiological investigations of community-acquired pneumonia in children admitted at the emergency department of a university hospital. *Journal of Clinical Virology*.

[32] Canducci F., Debiaggi M., Sampaolo M. (2008). Two-year prospective study of single infections and co-infections by respiratory syncytial virus and viruses identified recently in infants with acute respiratory disease. *Journal of Medical Virology*.

[33] Xepapadaki P., Psarras S., Bossios A. (2004). Human metapneumovirus as a causative agent of acute bronchiolitis in infants. *Journal of Clinical Virology*.

[34] van Woensel J. B. M., Bos A. P., Lutter R., Rossen J. W. A., Schuurman R. (2006). Absence of human metapneumovirus co-infection in cases of severe respiratory syncytial virus infection. *Pediatric Pulmonology*.

[35] Jin Y., Zhang R.-F., Xie Z.-P. (2012). Newly identified respiratory viruses associated with acute lower respiratory tract infections in children in Lanzou, China, from 2006 to 2009. *Clinical Microbiology and Infection*.

[36] Kuypers J., Martin E. T., Heugel J., Wright N., Morrow R., Englund J. A. (2007). Clinical disease in children associated with newly described coronavirus subtypes. *Pediatrics*.

[37] Gaunt E. R., Hardie A., Claas E. C. J., Simmonds P., Templeton K. E. (2010). Epidemiology and clinical presentations of the four human coronaviruses 229E, HKU1, NL63, and OC43 detected over 3 years using a novel multiplex real-time PCR method. *Journal of Clinical Microbiology*.

[38] Abed Y., Wang D., Boivin G. (2007). WU polyomavirus in children, Canada. *Emerging Infectious Diseases*.

[39] Bialasiewicz S., Whiley D. M., Lambert S. B. (2008). Presence of the newly discovered human polyomaviruses KI and WU in Australian patients with acute respiratory tract infection. *Journal of Clinical Virology*.

[40] Han T. H., Chung J. Y., Koo J. W., Kim S. W., Hwang E.-S. (2007). WU polyomavirus in children with acute lower respiratory tract infections, South Korea. *Emerging Infectious Diseases*.

[41] Le B. M., Demertzis L. M., Wu G. (2007). Clinical and epidemiologic characterization of WU polyomavirus infection, St. Louis, Missouri. *Emerging Infectious Diseases*.

[42] Debiaggi M., Canducci F., Ceresola E. R., Clementi M. (2012). The role of infections and coinfections with newly identified and emerging respiratory viruses in children. *Virology Journal*.

[43] Zhuang W. L., Lu X. D., Lin G. Y. (2011). WU polyomavirus infection among children in South China. *Journal of Medical Virology*.

[44] Rao S., Garcea R. L., Robinson C. C., Simões E. A. F. (2011). WU and KI polyomavirus infections in pediatric hematology/oncology patients with acute respiratory tract illness. *Journal of Clinical Virology*.

[45] Christensen A., Nordbø S. A., Krokstad S., Rognlien A. G. W., Døllner H. (2008). Human bocavirus commonly involved in multiple viral airway infections. *Journal of Clinical Virology*.

[46] Schildgen O., Müller A., Allander T. (2008). Human bocavirus: passenger or pathogen in acute respiratory tract infections?. *Clinical Microbiology Reviews*.

[47] Söderlund-Venermo M., Lahtinen A., Jartti T. (2009). Clinical assessment and improved diagnosis of bocavirus-induced wheezing in children, Finland. *Emerging Infectious Diseases*.

[48] Jartti T., Hedman K., Jartti L., Ruuskanen O., Allander T., Söderlund-Venermo M. (2012). Human bocavirus—the first 5 years. *Reviews in Medical Virology*.

[49] de Paulis M., Gilio A. E., Ferraro A. A. (2011). Severity of viral coinfection in hospitalized infants with respiratory syncytial virus infection. *Jornal de Pediatria*.

[50] Aberle J. H., Aberle S. W., Pracher E., Hutter H. P., Kundi M., Popow-Kraupp T. (2005). Single versus dual respiratory virus infections in hospitalized infants: impact on clinical course of disease and interferon-*γ* response. *Pediatric Infectious Disease Journal*.

[51] Spann K. M., Tran K. C., Chi B. (2004). Suppression of the induction of alpha, beta, and lambda interferons by the NS1 and NS2 proteins of human respiratory syncytial virus in human epithelial cells and macrophages. *Journal of Virology*.

